# Veia cava superior esquerda persistente: relato de caso

**DOI:** 10.1590/1677-5449.002815

**Published:** 2016

**Authors:** Mário Vinícius Angelete Alvarez Bernardes, Ronald Kool, Ivan Neutzling Lüdtke, Murilo de Almeida Luz, Fabiano Luiz Erzinger

**Affiliations:** 1 Hospital Erasto Gaertner – HEG, Residência Médica de Cirurgia Oncológica, Curitiba, PR, Brasil.; 2 Hospital Erasto Gaertner – HEG, Serviço de Cirurgia Abdominal, Curitiba, PR, Brasil.; 3 Hospital Erasto Gaertner – HEG, Serviço de Cirurgia Vascular, Curitiba, PR, Brasil.; 4 Instituto da Circulação, Curitiba, PR, Brasil.

**Keywords:** veia cava superior, anormalidades congênitas, malformações vasculares, cateteres, quimioterapia

## Abstract

O pleno conhecimento da anatomia vascular torácica é de suma importância para os profissionais envolvidos na realização de procedimentos invasivos como a punção de acesso venoso central. A persistência da veia cava superior esquerda é a malformação venosa torácica mais frequente, e seu diagnóstico costuma ser incidental. Apresentamos o caso de uma paciente de 14 anos em que o diagnóstico de veia cava superior esquerda persistente foi incidental em exame de imagem de controle após colocação de cateter venoso totalmente implantável. A paciente não apresentou dificuldade de infusão de quimioterapia pelo cateter e não houve complicações relacionadas ao cateter.

## INTRODUÇÃO

Dispositivos como o cateter totalmente implantável (CTI) de longa permanência são utilizados com frequência em pacientes oncológicos, em especial devido à sua praticidade para a infusão endovenosa de quimioterápicos. Os benefícios associados ao CTI devem sempre ser contrabalanceados com as complicações associadas ao procedimento^1^.

O pleno conhecimento da anatomia vascular torácica e cervical é de especial interesse para cirurgiões vasculares, cirurgiões oncológicos, radiologistas intervencionistas e demais médicos envolvidos no manejo de pacientes oncológicos, que por vezes necessitam realizar procedimentos para a aquisição de acesso venoso profundo^2^.

A persistência da veia cava superior esquerda (VCSEP) representa a anomalia congênita venosa torácica mais frequente, e seu conhecimento é crítico para o sucesso do implante de dispositivos invasivos, visando minimizar os riscos de potenciais complicações^3^.

Este artigo relata um caso de implante de CTI em posição anômala do lado esquerdo no mediastino.

## DESCRIÇÃO DO CASO

**Figura 1:**
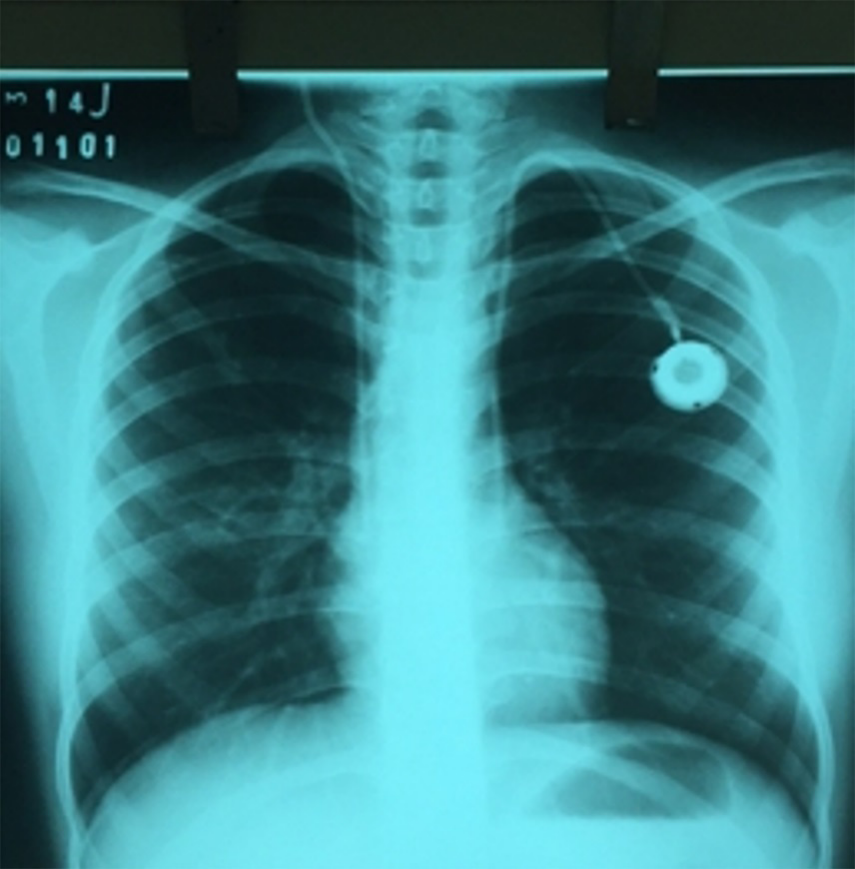
Radiografia de tórax de controle em incidência póstero-anterior após instalação do cateter totalmente implantável.

Paciente feminina, 14 anos, com história prévia de Síndrome de Turner e em vigência de tratamento quimioterápico por leucemia mieloide aguda através de cateter venoso central duplo lúmen em veia jugular interna direita. Devido indicação de utilização de dispositivo permanente para infusão de quimioterápico, foi submetida a colocação de CTI através de punção da veia jugular interna esquerda. Em exame radiológico de controle imediato pós-procedimento, o CTI foi identificado à esquerda do mediastino e não foram evidenciadas outras alterações (Figura 1).

O cateter apresentava bons fluxo e refluxo. Gasometria de amostra de sangue coletada pelo cateter confirmou tratar-se de sangue venoso.

Foi solicitada avaliação da Equipe de Cirurgia Vascular, que realizou angiografia venosa através da injeção de contraste diretamente pelo CTI, com achado compatível de VCSEP com drenagem para o átrio direito.

**Figura 2:**
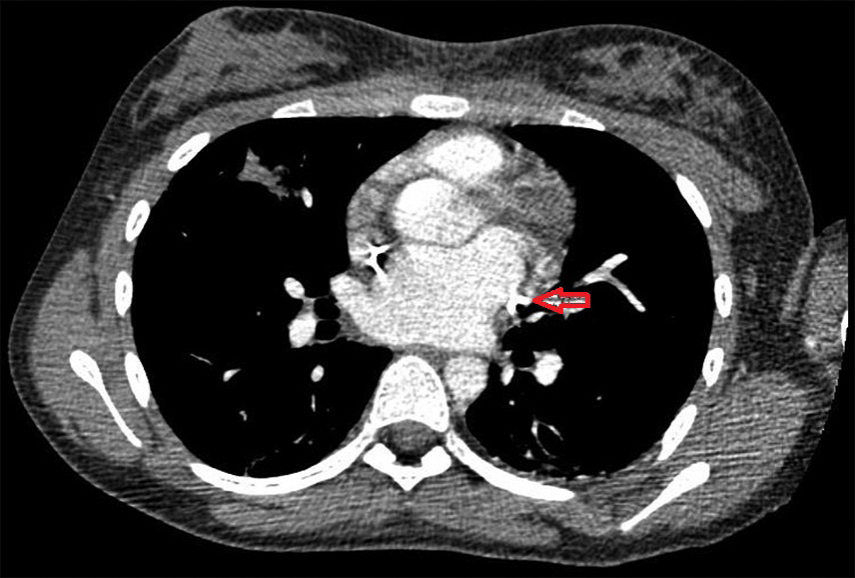
Angiotomografia computadorizada do tórax com evidência de veia cava superior esquerda persistente (seta).

A paciente foi submetida então à realização de ecocardiograma transtorácico, com evidência de aumento do diâmetro do átrio direito e sem outras anomalias cardíacas concomitantes. Angiotomografia computadorizada do tórax (Figura 2) realizada em sequência confirmou o diagnóstico de VCSEP.

Optou-se por manter o CTI para que a paciente pudesse dar continuidade ao tratamento quimioterápico proposto, com plano de seguimento ambulatorial e reavaliação quanto à retirada do cateter em momento oportuno.

A paciente encontra-se com 12 meses de seguimento clínico, sem sinais de recidiva da doença oncológica de base, assintomática do ponto de vista cardiovascular e sem sinais de complicações locais pelo CTI.

## DISCUSSÃO

**Figura 3:**
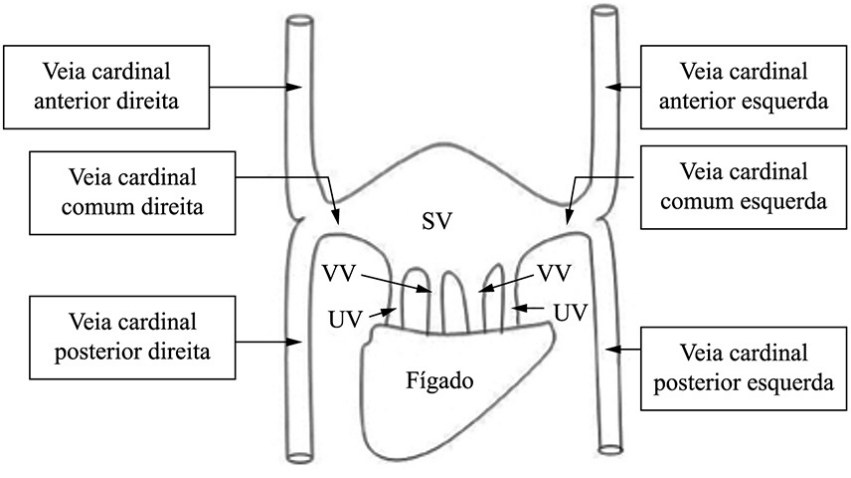
Desenho esquemático das veias cardinais e suas relações na fase embrionária (adaptado de: Singh^4^) SV: seio venoso; VV: veias vitelinas; UV: veias umbilicais.

Durante a 5ª semana de vida intrauterina, os fetos humanos possuem três veias principais: (a) a veia vitelina, que leva o sangue do saco vitelínico ao seio venoso; (b) as veias umbilicais, que se originam das vilosidades coriônicas e que trazem o sangue oxigenado para o embrião; e (c) as veias cardinais, responsáveis pela drenagem de sangue dentro do próprio embrião (Figura 3). As veias cardinais, por sua vez, são divididas em veias cardinais anteriores, responsáveis pela drenagem da porção cefálica do embrião, e veias cardinais posteriores, que drenam o restante do embrião. As veias cardinais drenam conjuntamente no seio coronário através de um canal cardinal comum^5^.

A VCSEP é uma anormalidade causada pela persistência da veia cardinal comum esquerda. Sua incidência varia entre 0,3% a 0,5% na população normal, podendo atingir até 10% dos pacientes sabidamente portadores de outra anomalia congênita cardíaca^6^. Em pacientes com Síndrome de Turner, essa incidência pode variar de 5 a 13%^7^^,^ 8.

A real incidência da VCSEP pode ser subestimada devido à não identificação dessa anomalia em pacientes assintomáticos e sem alterações cardíacas associadas. Na maioria dos casos a veia cava superior direita está presente, sendo raro o achado isolado de VCSEP. Casos em que a veia cava superior direita está ausente são mais frequentemente associados à ocorrência de outras anomalias cardíacas sincrônicas^9^. Em trabalho retrospectivo, Cha e Khoury revisaram 275 angiografias cardíacas e encontraram 12 casos de VCSEP, dentre os quais apenas dois não apresentavam a veia cava superior direita^6^. Em outro estudo, Iovino et al.^10^, ao analisarem 600 pacientes submetidos à colocação de CTI, encontraram quatro casos de VCSEP, sendo que em 85% dos casos o sítio de punção escolhido foi a veia jugular interna esquerda. Em ambas séries não houve complicações relacionadas ao cateter.

A evidência na radiografia de tórax de contorno anormal do mediastino superior à esquerda decorrente do alargamento da sombra da aorta e a proeminência paramediastinal abaixo do arco da aorta são comumente associadas à VCSEP^11^. A presença de uma faixa estreita de menor densidade ao longo da borda cardíaca superior esquerda ou a existência de sombra em crescente que se estende da borda superior esquerda do arco aórtico até o terço médio da clavícula^12^ também levantam a suspeita de VCSEP. A dilatação do seio coronário observada ao ecocardiograma corrobora o diagnóstico^13^.

Na ocorrência de atresia da veia inominada esquerda, deve-se sempre aventar a hipótese de VCSEP. Por sua vez, essa anomalia requer uma modificação na técnica cirúrgica adotada em cirurgias cardíacas com circulação extracorpórea, durante a qual a VCSEP deve ser canulada separadamente. Em pacientes com atresia tricúspide submetidos a tratamento cirúrgico através da cirurgia de Glenn, na qual é realizada anastomose término-lateral entre a veia cava superior e a artéria pulmonar direita, deve-se adicionar uma anastomose término-lateral entre a veia cava superior esquerda e a artéria pulmonar esquerda, sendo essa modificação técnica conhecida como operação de Kawashima^14^.

O achado através do ecocardiograma transtorácico de dilatação isolada do átrio direito pode ser justificado pela presença de VCSEP com drenagem anômala no átrio direito através do seio coronário. A tomografia computadorizada com contraste, por sua vez, ao permitir a identificação da veia cava superior esquerda no mediastino superior esquerdo adjacente à artéria carótida e anterior à artéria subclávia, mantendo-se lateralmente ao arco da aorta e ao tronco da artéria pulmonar, passando anteriormente ao hilo esquerdo e finalmente desaparecendo na silhueta cardíaca, também tem sido de grande utilidade no diagnóstico dessa anomalia da VCSEP^15^.

De maneira geral, o diagnóstico de VCSEP é incidental, em especial após a passagem de acesso venoso central ou implante de marca-passo inseridos através da punção das veias jugular interna e subclávia esquerdas, estando o cateter localizado à esquerda do mediastino em exame radiográfico de controle^16^^-^ 18.

Em 90% dos pacientes portadores de VCSEP, esta desemboca no átrio direito através do seio coronário, enquanto que os 10% restantes drenam diretamente para o átrio esquerdo, onde o seio coronário estará ausente^19^^,^ 20.

Apesar de geralmente assintomática, essa anomalia congênita venosa pode tornar o paciente mais susceptível a arritmias cardíacas devido a alterações no nó atrioventricular e no feixe de His. O prejuízo do fluxo atrioventricular esquerdo devido à obstrução parcial da válvula mitral também pode ser observado como sintomatologia nos pacientes portadores dessa anomalia venosa. Entre os pacientes portadores de VCSEP e cuja drenagem faz-se diretamente para o átrio esquerdo, pode ainda ocorrer *shunt* da direita para a esquerda^21^.

Em procedimentos de colocação de CTI, deve-se sempre ter a preocupação quanto à localização do cateter, em especial em pacientes oncológicos e que requerem a infusão de quimioterápicos e substâncias hiperosmolares. Devido à escassez de dados na literatura que suportem a infusão dessas medicações através de cateteres com dimensão inferior a 7 Fr (2,3 mm), deve-se evitar a utilização de dispositivos inseridos em veias de menor calibre.

É primordial ressaltar ainda a importância de que um paciente diagnosticado como portador de uma anomalia congênita como a VCSEP seja submetido a investigação complementar para excluir a existência de anomalias congênitas concomitantes, uma vez que essa anomalia pode estar associada à existência de defeitos do septo interatrial e defeitos da drenagem venosa^15^.
