# Supplementary material for: Veia cava superior esquerda persistente: relato de caso
Source: J Vasc Bras. 2016 Apr-Jun;15(2):153–7. [Article in Portuguese] doi: 10.1590/1677-5449.002815 (PMC5829711; doi:10.1590/1677-5449.002815)
Supplement: Translation

# Translation: Persistent left superior vena cava: case report

**Authors:** Mário Vinícius Angelete Alvarez Bernardes, Ronald Kool, Ivan Neutzling Lüdtke, Murilo de Almeida Luz, Fabiano Luiz Erzinger

**Affiliations:** 1 Hospital Erasto Gaertner – HEG, Residency of Surgical Oncology, Curitiba, PR, Brazil.; 2 Hospital Erasto Gaertner – HEG, Department of Abdominal Surgery, Curitiba, PR, Brazil.; 3 Hospital Erasto Gaertner – HEG, Department of Vascular Surgery, Curitiba, PR, Brazil.; 4 Instituto da Circulação, Curitiba, PR, Brazil.

**Keywords:** superior vein cava, congenital abnormalities, vascular malformations, catheters, chemotherapy

## Abstract

It is extremely important that health professionals involved in invasive procedures such as central venous access have a thorough knowledge of thoracic vascular anatomy. Persistent left superior vena cava is the most common congenital thoracic venous abnormality and is generally diagnosed as an incidental finding. We present the case of a 14-year-old female patient in whom a persistent left superior vena cava was diagnosed as an incidental finding of a control imaging exam after placement of a totally implantable venous catheter. The patient did not exhibit any difficulties with infusion of chemotherapy via the catheter and there were no complications related to the catheter.

## INTRODUCTION

Devices such as long-term totally implantable catheters (TIC) are often used in cancer patients, particularly because of their practicality for intravenous infusion of chemotherapy. The benefits associated with TIC must always be balanced against the complications associated with the procedure.^1^ A thorough knowledge of thoracic and cervical vascular anatomy is extremely important for vascular surgeons, surgical oncologists, interventional radiologists and other physicians involved in management of oncology patients who sometimes need to perform procedures to obtain deep venous access.^2^

Persistent left superior vena cava (PLSVC) is the most common congenital thoracic venous anomaly and knowing about it is critical to the success of implanting invasive devices, in terms of minimizing the risks of potential complications.^3^

This article describes a case in which a TIC was implanted in an anomalous position on the left side of the mediastinum.

## CASE DESCRIPTION

The patient, a 14-year-old female with a prior history of Turner Syndrome, was receiving chemotherapy to treat acute myeloid leukemia via a double lumen central venous catheter placed via the right jugular vein. She had been prescribed a permanently implanted device for chemotherapy infusion and underwent placement of a TIC via puncture of the left internal jugular vein. A control X-ray examination conducted immediately after the procedure located the TIC in the left mediastinum, with no evidence of other abnormalities (Figure 1).

**Figure 1:** Control posteroanterior chest X-ray after placement of the totally implantable cateter.

The catheter exhibited good flow and reflux and blood gas analysis of a sample taken through the catheter confirmed that it was venous blood.

The Vascular Surgery team were asked to conduct an assessment and they performed a venous angiography with injection of contrast directly through the TIC and reported findings compatible with a PLSVC draining into the right atrium.

A transthoracic echocardiogram showed evidence of an enlarged right atrial diameter, but no other concomitant cardiac anomalies. Computed tomography angiography of the thorax was then performed (Figure 2), confirming the diagnosis of PLSVC.

**Figure 2:** Computed tomography angiography of the chest showing evidence of a persistent left superior vena cava (arrow).

It was decided not to remove the TIC, to allow the patient to complete the proposed course of chemotherapy treatment, and outpatients follow-up and reassessment were planned for when the catheter was removed at the appropriate time.

At the time of writing, the patient has been in clinical follow-up for 12 months with no signs of relapse of the underlying oncological disease, she is asymptomatic from a cardiovascular point of view and free from signs of local complications secondary to the TIC.

## DISCUSSION

During the 5th week of intrauterine life, human fetuses have three principal veins: (a) the vitelline vein, that takes blood from the vitelline sac to the sinus venosus; (b) the umbilical veins, that arise in the chorionic villosities and bring oxygenated blood to the embryo; and (c) the cardinal veins, responsible for drainage of blood within the embryo itself (Figure 3). In turn, the cardinal veins are divided into the anterior cardinal veins, responsible for drainage of the cephalic portion of the embryo, and the posterior cardinal veins, which drain the remainder of the embryo. The cardinal veins drain into the coronary sinus together, via a common cardinal vein.^5^

**Figure 3:** Drawing illustrating the cardinal veins and their connections during the embryonic phase (adapted from: Singh^4^) SV, sinus venosus; VV, vitelline veins; UV, umbilical veins.

PLSVC is an abnormality caused by persistence of the left common cardinal vein. Incidence varies from 0.3% to 0.5% of the normal population, although it is found in as many as 10% of patients known to have other congenital cardiac anomalies.^6^ Incidence among patients with Turner Syndrome, can range from 5 to 13%.^7^^,^ 8

The true incidence of PLSVC may be underestimated because of failure to identify the anomaly in asymptomatic patients and those with no associated cardiac abnormalities. In the majority of cases, the right superior vena cava is present and it is rare to find a PLSVC in isolation. Cases in which the right superior vena cava is absent are associated with occurrence of other cardiac anomalies is increased.^9^ Cha and Khoury conducted a retrospective study reviewing 275 cardiac angiographs and found 12 cases of PLSVC, of which only two did not have a right superior vena cava.^6^ In another study, Iovino et al.^10^ analyzed 600 patients who had TICs fitted, finding four cases of PLSVC, and in 85% of these cases the puncture site chosen was the left internal jugular vein. There were no complications related to the catheters in either of these series.

Evidence on a chest X-ray of an abnormal outline to the superior left mediastinum caused by an expansion of the shadow of the aorta and a paramediastinal prominence below the arch of the aorta are often associated with a PLSVC.^11^ Presence of a narrow band of lower density along the superior left cardiac margin or a crescent-shaped shadow extending from the superior left margin of the aortic arch to the mid third of the clavicle should also raise a suspicion of PLVSC.^12^ Dilation of the coronary sinus on an echocardiogram confirms the diagnosis.^13^

Atresia of the left brachiocephalic vein should always suggest a hypothesis of PLSVC. When present, the anomaly may demand modification to the surgical technique adopted for cardiac surgery with extracorporeal circulation, during which the PLSVC should be cannulated separately. For patients with tricuspid atresia treated with Glenn surgery, in which end-to-side anastomosis of the superior vena cava to the right pulmonary artery is performed, an end-to-side anastomosis of the left superior vena cava to the left pulmonary artery should be added, and this modification to the technique is known as the Kawashima procedure.^14^

A finding of isolated dilation of the right atrium on a transthoracic echocardiogram may be explained by a PLSVC with anomalous drainage into the right atrium via the coronary sinus. In turn, computed tomography with contrast is also of great utility for diagnosis of the PLSVC anomaly since it allows identification of the left superior vena cava in the left superior mediastinum, adjacent to the carotid artery and anterior of the subclavian artery, remaining lateral to the arch of the aorta and the trunk of the pulmonary artery, passing anteriorly of the left hilum and finally disappearing in the cardiac silhouette.^15^

In general, PLSVC is diagnosed incidentally, particularly after placement of a central venous access or a pacemaker inserted via puncture of the left internal jugular and subclavian veins, when the catheter will be seen on the left of the mediastinum on a control X-ray.^16^^-^ 18

In 90% of patients with a PLSVC, it drains into the right atrium through the coronary sinus, whereas in the remaining 10% of cases it drains directly into the left atrium, where the coronary sinus will be absent.^19^^,^ 20

Although this congenital venous anomaly is generally asymptomatic, it can make patients more susceptible to cardiac arrhythmia because of abnormalities to the atrioventricular node and the bundle of His. Compromised left atrioventricular flow caused by partial obstruction of the mitral valve can also be observed among the symptoms of patients with this venous anomaly. Patients with PLSVC and drainage directly into the left atrium may also exhibit right-to-left shunt.^21^

In procedures for placement of a TIC, care should always be taken with the location of the catheter, especially in cancer patient who will need infusion of chemotherapy drugs and hyperosmolar substances. Considering the scarcity of data supporting infusion of these medications via catheters with dimensions smaller than 7 Fr (2.3 mm), use of devices inserted into smaller caliber veins should be avoided.

It is extremely important to emphasize that when a patient is diagnosed with a congenital anomaly such as PLSVC, a supplementary investigation should be conducted to rule out concomitant congenital anomalies, since this anomaly can be associated with defects of the interatrial septum and venous drainage defects.^15^
